# A stable CC-chemokine receptor (CCR)-5 tropic virus is correlated with the persistence of HIV RNA at less than 2.5 copies in successfully treated naïve subjects

**DOI:** 10.1186/1471-2334-13-314

**Published:** 2013-07-11

**Authors:** Saverio Giuseppe Parisi, Samantha Andreis, Carlo Mengoli, Renzo Scaggiante, Mario Cruciani, Roberto Ferretto, Vinicio Manfrin, Sandro Panese, Monica Basso, Caterina Boldrin, Stefania Bressan, Loredana Sarmati, Massimo Andreoni, Giorgio Palù

**Affiliations:** 1 Department of Molecular Medicine, University of Padova, Via Gabelli 63, 35100, Padova, Italy; 2 Center of Preventive Medicine & HIV Outpatient Clinic, Verona, Italy; 3 Infectious Diseases, SchioHospital, Schio, Italy; 4 Infectious Diseases, VeneziaHospital, Venezia, Italy; 5 Clinical Infectious Diseases, TorVergataUniversity, Rome, Italy

**Keywords:** HIV tropism, HIV residual viraemia, HIV DNA load

## Abstract

**Background:**

To determine if tropism for CXCR4 or CCR5 correlates with cellular HIV DNA load, residual viraemia and CD4 count in 219 successfully treated naive subjects with HIV infection enrolled in five infectious diseases units in Northeastern Italy.

**Methods:**

A subset of subjects, achieving plasma HIV RNA level <50 copies/ml after initiation of first-line therapy and maintaining it until follow-up time points, was retrospectively selected from a prospective cohort. Blood samples were collected before the beginning of therapy (T0), at the first follow-up time (T1) and, when available, at a second (T2) follow-up time.

**Results:**

HIV DNA, CD4 count and plasma viraemia were available from all 219 patients at T0 and T1, and in 86 subjects at T2, while tropism determinations were available from 109 subjects at T0, 219 at T1, and from 86 subjects at T2. Achieving residual viraemia <2.5 copies/ml at T1 correlated with having the same condition at T2 (p = 0.0007). X4 tropism at T1 was negatively correlated with the possibility of achieving viraemia<2.5 copies/ml at T2 (p = 0.0076). T1-T2 tropism stability was significant (p <0.0001). T0 tropism correlated with T1 and T2 tropism (p < 0.001); therefore the stability of the tropism over the two follow-up periods was significant (p = 0.0003). An effective viremic suppression (viraemia<2.5 copies/ml) correlated with R5 coreceptor affinity (p= 0.047).

**Conclusions:**

The tropism of archived virus was stable during an effective treatment, with 15-18% of subjects switching over time, despite a viraemia<50 copies/ml. R5 tropism and its stability were related to achieving and maintaining viraemia<2.5 copies/ml.

## Background

Several chemokine receptors are coreceptors for HIV entry into cells. Non-syncytium-inducing HIV variants use the CC-chemokine receptor (CCR)-5 as a coreceptor (R5 viruses) [1-4], whereas syncytium-inducing HIV variants use the CXC-chemokine receptor (CXCR)-4 (X4 viruses) and, in some cases, sometimes, in addition, CCR-5 (dual/mixed or D/M viruses) [5]. The co-receptors CCR5 and CXCR4 are both expressed in the cell and tissue targets of HIV, consistent with their roles in disease transmission and progression [6]. The R5 viruses are generally predominant in the early stages of HIV infection, whereas the emergence of X4 viruses generally occurs in the later stage [7-9]. The presence of X4 viruses is consistently associated with low CD4 T-cell counts and accelerated disease progression, although it is unclear whether the presence of X4 viruses is a cause or consequence of disease progression [6,7].

The dynamics and the influence of viral tropism on the course of HIV-1 infection in subjects exposed to antiretroviral therapy (ARV) are not fully understood. Several studies have demonstrated a higher prevalence of X4 variants in patients exposed to antiretroviral drugs than in drug-naïve individuals [10,11]. The studies that have attempted to correlate viral tropism with the response to ARV have generally demonstrated no differences in the response to treatment between patients harbouring an X4- or R5-tropic virus, although a poor recovery in the number of CD4+ cells has been shown in patients with an X4-tropic virus before the beginning of treatment or in PBMCs (peripheral blood mononuclear cells) after treatment [1,12,13]. The presence of an X4-tropic virus has been demonstrated to have a deleterious effect on CD4 cell count growth and the risk of clinical disease in patients undergoing the first line of HAART (Highly Active Antiretroviral Therapy) [7].

Studies aimed at assessing the influence and frequency of the switch of viral tropism during the course of therapy showed a low event rate in patients responding to HAART, and there have been conflicting results regarding the response to treatment, even if a direct correlation between the switch from an R5- to an X4-tropic virus and a low CD4 + nadir was found in treated patients [14-17].A change in viral tropism has been shown to occur in patients during ARV interruption, and the switch from an R5-tropic to an X4-tropic virus is associated with a previous heavy treatment experience, a considerable decrease in the CD4 cell count and a shorter length of treatment interruption [18,19].

Up to now, there are few data from longitudinal studies on viral tropism in patients fully responding to HAART, and in particular, there are insufficient data on the correlation between viral tropism and combined HIV-proviral DNA, residual viraemia and drugs used in treatment.

To assess the impact of antiretroviral therapy on viral tropism evolution and the correlations between tropism and the response to therapy in terms of HIV DNA and residual viraemia values, we retrospectively examined a group of 219 HIV-1-infected naïve individuals who were successfully treated with ARV.

## Methods

### Study design

The CAVeAT is a prospective cohort of HIV-infected patients enrolled starting from 2004 in five infectious diseases units in Northeastern Italy (Veneto region). We retrospectively selected from this cohort of patients a subset of subjects achieving virological suppression within 6 months after initiation of first-line therapy and maintaining plasma HIV RNA levels < 50 copies/ml, without virological failures, until evaluation at the follow-up time points. Patients interrupting or needing treatment modifications for failure, as well as patients who had more than one viral blip (plasma HIV RNA from 50 to 1.000 copies/ml) per year after achieving viral suppression, which was determined by frequent blood sampling (at least 4 times per year), were not included in the study. In the event of a change in the HAART, it was accepted if this change was due to intolerance or pill burden convenience. No patient was treated with CCR5 antagonists.

Blood samples were collected at baseline (before the beginning of therapy), at the first follow-up time (T1) and, when available, at a second (T2) follow-up time.First follow up time (T1) was defined as the first available time point after achieving virological suppression within a range from 12 to 72 months, and T2 as a more recent time point available, collected a median time of 16 months after T1. All virological and immunological analyses were performed on all samples, with the exception of the tropism analysis, which was performed on all samples available at T1 and T2 but on only 109 out of 219 subjects at T0 due to depletion of the samples. This cohort was initially enrolled for another study on DNA load [20].

The enrolled subjects gave informed consent for all procedures and the use of their blinded data for a scientific evaluation and publication. The local government, which was represented by the Veneto Regional Health Authority, approved the study and provided funding (Regional Government Decree 3643/2004 and 3499/2008). This study was conducted in accordance with the Helsinki Declaration and local legislation (Ethics Committee of Padova University Hospital prot. 2606-12P).

A primary HIV infection was defined by (i) a negative or indeterminate HIV antibody enzyme linked immunosorbent assay associated with HIV RNA positive plasma or (ii) an initially negative test for HIV antibodies followed by a positive serology within 18 months.

The blood samples were collected in tubes containing EDTA and separated into plasma and cells using Ficoll-Paque Plus density gradient centrifugation. Aliquots of plasma and dried pellets of 2 × 10^6^ PBMCs were stored at −80°C until use. Baseline and follow-up blood samples in EDTA were submitted within 6 hours from collection to the Laboratory of Virology at the University of Padova and stored until analysis.

### Cellular HIV DNA quantitation

The extraction and purification of DNA from cells were performed using the QIAmp Blood kit (Qiagen, Inc., Chatsworth, CA). The real-time TaqMan protocol was used to quantify the cellular HIV DNA copy number in PBMCs as previously described [20]. The cell line 8E5, which contains one copy of integrated HIV DNA in each cell, was used to build a standard curve with a sensitivity of 5 copies/million PBMCs [21].

A duplicate analysis within the same experiment was conducted for both HIV and beta-globin evaluation, and the mean of these values was calculated. When the delta values were more than 10%, the sample was analysed again. Baseline and follow-up samples from a patient were always evaluated in the same experiment; this procedure was possible because only complete sample sets (T0 and T1, and T2 when available) of the patients, retrospectively selected, were considered. A sample that had been previously evaluated was regularly added as an internal control.

### Quantification of plasma residual viraemia

The plasma samples were obtained from the blood that was collected and were frozen at −80°C within 6 hours of collection and kept at this temperature until testing.

Residual viraemia was quantified, using an ultra-ultrasensitive method based on a modified Amplicor HIV-1 Monitor test, version 1.5 (Roche Molecular Systems, Branchburg, New Jersey, USA), with a limit of detection limit of 2.5 copies/ml [20]. Modifications included pelleting viruses from 2 ml of plasma at 23.600 g at 4°C for 2 hours, adding half of the normal volume of the quantification standard and resuspending the RNA pellet in 50 ul of diluent. The entire volume of the resuspended RNA was assayed, using reverse transcription and PCR amplification. These and subsequent detection steps followed were performed according to the manufacturer’s protocol.

### Genotypic prediction of viral tropism

Genotypic analysis of viral tropism was performed on PBMCs as previously described [22]. Briefly, the V3 sequences were amplified using nested PCR with 1F1 and 1R1 as the outer primers and 3F3 and 2R2 as the inner primers.We checked the number of ambiguities examined before running geno2pheno: they were considered relevant if the number was greater than two. In that case the sequencing was repeated and the sample was excluded if the ambiguities were confirmed. Seven samples met this criteria and the seven patients were not included. When a single or double ambiguity gave discordant results in terms of FPR, sequencing was repeated.

The generated V3 sequences were then interpreted using the bioinformatic tool Geno2pheno, with a false-positive rate of 20%. Geno2pheno is available at http://coreceptor.bioinf.mpi-inf.mpg.de (accessed by June 2012).

All PBMCs available from the subjects gave a useful amplification and sequencing of the V3 region, allowing us to analyse all subjects.

All longitudinal discordant results were analysed twice, to confirm the tropism switch; similarly, all samples with an false positive rate from 10% to 30% were confirmed by a second analysis starting from the amplification of the sample. In summary, we determined and described a clear viral switch only with two concordant predictions.

### Statistical analysis

The following variables were analysed: age, CD4 cell count at T0 and follow-up, and HIV DNA log_10_ copies/10^6^ PBMCs at T0 and follow-up. The HIV RNA log_10_ copies/ml was evaluated as continuous variable. HIV RNA at follow-up under the lowest threshold level (under 2.5 copies/ml), primary HIV infection at the start of therapy, stranger origin, male gender, risk categories (heterosexual, men who have sex with men, drug addiction) and tropism based on the presence of R5 or X4 variants in PBMCs at T0, T1 and T2 were evaluated as binary variables.

All variables were submitted to pairwise correlation analysis to outline the underlying link pattern in the evaluated population. The correlation was established by a linear regression analysis when at least one variable was continuous. When both paired variables were categorical (binary), the chi-square test by Pearson or the Fisher’s exact test (when applicable) were used.We performed a further statistical analysis based on X4 detection and R5 detection as binary variable and the patient as grouping variable (most patients had repeated observations). Unifying the time-points under a common time scale, a multilevel/mixed logistic regression was performed, using plasma RNA under low threshold as outcome, and the following covariates: log10DNA, baseline log10RNA, X4R5 (binary variable), and time. Conventional descriptive statistics were also applied when appropriate.

## Results

### Tropism analysis at T1

The sequences from the PBMCs of 219 patients were genotyped at a median follow-up of 36 months (range 12–72) after the initiation of therapy. The demographic, virological and immunological data are reported in Table 1.

**Table 1:** **Demographic**, **virological and immunological data for the entire population and the R5 and X4 subgroups**, **as identified by genotyping at T1**

	**All patients**	**R5 subgroup patients**	**X4 subgroup patients**	**p**
Number	219	146	73	ns
Male (n)	173	118	55	ns
Italian (n)	180	114	66	ns
Age^a^ (years)	41 (19–79)	40 (19–74)	43 (21–79)	p= 0.0332
*Risk*^*b*^				
Heterosexual (n)	91	60	31	ns
MSM (n)	67	47	20	ns
Drug user (n)	18	11	7	ns
Missing (n)	43	28	15	ns
Subtype-B (n)	171	113	56	ns
Primary^c^n (%)	29 (13.2%)	21 (14.3%)	8 (11%)	Ns
T0 CD4 cells/mm^3a^	260 (0–1090)	284 (0–810)	201 (7–1090)	p= 0.0216
T0 CD4%^a^	16 (0–39)	17 (0–37)	13 (1–39)	ns
T0 HIV RNA^a^ copies/ml	101836 (412–1x10^7^)	101836 (412–1x10^7^)	1000182 (547–6258236)	ns
T0 HIV DNA^a, d^	1198 (33–70123)	1185 (70–18856)	1297 (33–70123)	ns
T1 CD4 cells/mm^3a^	560 (54–1330)	580 (54–1306)	485 (96–1330)	ns
T1 CD4%^a^	28.9 (3.4–55.2)	29.9 (3.4–55,2)	26 (5–51.9)	ns
T1 HIV RNA<2,5 copies/ml n (%)	83 (37.9%)	62 (42.4%)	21 (28.7%)	ns
T1 HIV DNA^a, d^	107 (5–892)	105 (5–892)	110 (5–740)	ns

^a^Expressed as median and range.

^b^Transmission risk reported as prevalent.

^c^ Being in HIV Primary Infection at the start of therapy.

^d^ Copies/10^6^PBMCs.

Of the 219 subjects, 146 (66.6%) had an R5 genotype in PBMCs. Eighty-three of 219 subjects (37.9%) had < 2.5 copies/ml of HIV RNA at T1. In particular, 62 of 146 patients (42.4%) harbouring a R5-tropic virus and 21 of 73 patients (28.7%) with an X4 virus reached fewer than < 2.5 copies/ml of HIV RNA (p=ns). The analysis of viro-immunological parameters before starting HAART showed a significantly higher CD4 cell count at baseline in R5 than in X4 patients (median values 284 cells, range 0–810, vs. 201 cells, range 7–1090, p= 0.0216).

### Tropism analysis of patients with T0 and T1 evaluations

A tropism analysis of PBMCs at the baseline of therapy (T0) was available in 109 of 219 subjects from the study population.

At the T0, 75 subjects (69%) had R5 tropism. The subjects harbouring an X4-tropic virus had a lower baseline CD4 absolute count than the patients with an R5-tropic virus (p= 0.0012) and higher baseline plasma HIV RNA levels (p = 0.0431). With regard to the genotype stability, the results showed that at T1, after a median time of 24 months of follow up (range 12 – 48), most patients (89 out of 109, 81.6%,) maintained the same viral tropism. (Table 2): 12 out of 75 R5 subjects (16%) switched to X4.

**Table 2:** R5 and X4 tropism at T0 and T1

	**R5 T1 (n)**	**X4 T1 (n)**	**Total**
R5 T0 (n)	63	12	75
X4 T0 (n)	8	26	34
Total	71	38	109

(Persistence of the tropism: 82% Pearson chi2(1) = 37.6715, P<0.001). Eighty-nine subjects didn’t switch.

No differences in the follow-up duration were observed between the patients harbouring an R5- or an X4-tropic virus or between the switching or non-switching subjects (data not shown). The subjects with a stable X4 virus had a median CD4 cell gain of 235 cells (increased from 185 to 420 cells), and the patients with a stable R5 virus had a CD4 cell gain of 237 cells (from 305 to 542 cells). The patients who switched to an X4 virus presented a CD4 cell gain of 197 cells (from 228 to 425 cells), whereas the patients who switched to an R5 virus had a CD4 cell gain of 496 cells (from 104 to 600 cells). The patients with a stable X4 virus showed a decrease in HIV DNA from 516 to 58.5 copies/10^6^ PBMCs (median values), and the patients with a stable R5 virus showed a decrease in HIV DNA from 896 to 127 copies/10^6^ PBMCs.The patients who switched to an X4 virus showed a decrease in HIV DNA from 1432 to 159 copies/10^6^ PBMCs, and the patients who switched to an R5 virus showed a decrease in HIV DNA from 419.5 to 111 copies/10^6^ PBMCs.

At T1, 42.3% (11/26) of the patients with a stable X4 virus, 46% (29/63) of the patients with a stable R5 virus, 16.6% (2/12) of the patients who switched to an X4 virus and 43% (3/8) of the patients who switched to an R5 virus reached fewer than 2.5 copies/ml of HIV RNA (p = ns).

### Tropism analysis of patients with T1 and T2 evaluation

A second evaluation of HIV tropism and of viro-immunological parameters was obtained in 86/219 subjects at T2 (median time of 16 months after T1). An R5 tropic strain was detected in 5 patients (67%) (Table 3). In 73 out of 86 patients (p<0.0001) no modification of viral tropism occurred from the T1 to the T2 time point.

**Table 3:** DNA and CD4 evolution in 86 patients followed from the T1 to the T2 time point and detection of residual viraemia at T2

Patients (n)**	**Stable X4 (22)**	**Stable R5 (51)**	**Switch to X4 (6)**	**Switch to R5 (7)**
CD4 T1^a^ cells/mm^3^	431 (130 – 950)	560 (54 – 1170)	600 (330 – 860)	420 (315 – 890)
CD4 T2^a^ cells/mm^3^	520 (170 – 1110)	615 (92 – 1291)	540 (290 – 970)	535 (420 – 740)
HIV DNA T1^a^ Copies/10^6^PBMCs	41 (<5 – 657)	95 (<5 – 892)	75.5 (<5 – 532)	196 (18 – 424)
HIV DNA T2^a^ Copies/10^6^PBMCs	50.5 (<5 – 448)	83 (<5 – 469)	31 (<5 – 412)	70 (7 – 147)
HIV RNA <2,5 copies/ml (n/total n patients and percentage)^b^	2/22 (9.1%)	19/51 (37.2%)	4/6 (66.7%)	1/7 (14.3%)

^a^Expressed as median and range.

^b^ Pearson chi2 (3)=11.5664, *P* = 0.0009).

** The stability of the tropism (stable X4 plus stable R5 vs switch to R5 plus switch to X4) was significant p< 0.0001.

Six of the57 subjects harbouring an R5 virus at T1 switched to an X4 virus at T2, whereas seven of 22 patients with an X4 virus switched to an R5 virus at T2. No differences in the follow-up duration were observed among the four groups of patients. The ability to reach the lowest threshold of plasma HIV RNA at T1 correlated with having the same viral tropism at T2 (p = 0.0007). With respect to the correlation of residual viraemia with viral tropism, a condition of X4 tropism at T1 was negatively correlated with the possibility of reaching virological success with plasma RNA under the lowest threshold at the second time point (p= 0.0076). The relation among tropism evolution from T1 to T2 and the detection of plasma viremia lower than 2.5 copies/ml at T2 is reported in table 3 (p = 0.009). No differences in the therapy regimens were found among the groups (data not shown).

The mean CD4 cell increase from T1 to T2 was significantly higher for R5 harbouring patients (p = 0.0497).

Similarly, the persistence of HIV RNA less than 2.5 copies/ml at T1 and T2 was detected in non-switcher patients. In stable R5 patients, the persistence of an undetectable viral load was 76% (p = 0.002).

### Tropism analysis in patients with T0, T1 and T2 evaluations

In a subgroup of 51 patients, a viral tropism evaluation of the PBMCs at all 3 time points T0, T1 and T2 was available.The stability of the tropism over the long follow up, from T0 to T2, was significant (p = 0.0003).

Six of the 37 R5 patients at T0 switched to X4 at T2. In the evaluation of the sequence of the coreceptor tropism status of the patients at the three time points, 5 of the 8 subjects with plasma RNA levels below 2.5 copies/ml at T2 were stable R5, one was R5-R5-X4, one was R5-X4-R5, and one was X4-R5-X4 ( p= ns).

Twenty-four percent of patients that were stable R5 from T0 to T2 had less than 2.5 copies of HIV RNA, compared to none with a stable X4.

A significant persistence (p = 0.047) of the virological effect, defined as reaching the <2.5 copies RNA level at T1 and maintenance at T2, was noted in non-switcher patients with R5 virus.Overall, 9 R5 patients had undetectable viremia at T1 and 5 confirmed virological success at T2.An RNA level below the lowest threshold at T1 and T2 was found in subjects with persistence of the R5 status at the transition from T0 to T1. The persistence of the acquired RNA level was 73% (p = 0.047).

The baseline X4 condition was correlated with lower values of cellular HIV DNA, both at T1 (p= 0.0288) and at T2 (p= 0.0095). HIV DNA levels (p=0.002) and the condition of having a primary infection at the start of therapy (p= 0.008) were related to attaining RNA virological success, defined as having <2.5 copies/ml of HIV RNA.

#### Quality control

A total 47 patients had a FPR between 10-20%: 10 (9.2%) in the group of 109 patients evaluated at T0, 24 (11%) in the group of 219 patients with tropism available at T1 and 13 (15.1%) in the group of 86 patients studied at T2. Overall, 6 out of 21 (28.6%) patients with an FPR > 10% and 9/42 (21.4%) with an FPR < 10% switched to R5.

#### Multilevel/mixed logistic regression

The multilevel/mixed logistic regression confirmed that R5 is independently correlated to the achievement of plasma HIV RNA under 2.5 copies/ml.

Three covariates appeared negatively correlated to the favorable virological outcome: log10DNA, baseline log10RNA, X4R5 in a significant manner. The coefficients of the regression are reported in Table 4.

**Table 4:** **Mixed**-**effects logistic regression**

**Plasma RNA under low threshold**	**Coef.**	**Std. Err.**	**z**	**P>z**	**95% Conf. interval**	
log10 DNA	−1.6098	0.5018	−3.21	0.001	−2.5932	−0.6263
baseline log10 RNA	−0.6943	0.3273	−2.12	0.034	−1.3359	−0.0528
X4	−1.4030	0.5493	−2.55	0.011	−2.4795	−0.3265
Time	−0.0072	0.0128	−0.56	0.573	−0.0322	0.0178
Intercept	6.3031	2.1489	2.93	0.003	2.0913	10.5150

Group variable: patient. Log likelihood = −143.7488, Wald chi^2^(4) = 13.44, P = 0.0093.

## Discussion

This study is the first to analyse the relationship of tropism of archived virus with residual plasma viraemia below 2.5 copies/ml and HIV DNA cellular load in a cohort of naïve patients who underwent and mantained successful treatment, with a baseline and two follow-up time points. The observation time was prolonged, with a median of 36 months from the beginning of treatment and 16 months thereafter in a subgroup of patients.

The stability of tropism in 109 subjects from the beginning of therapy to a median follow-up point of 24 months was significant (p< 0.001), with 89 subjects (82%) remaining in the same condition as that at the baseline, 8 subjects switching to R5 and 12 subjects switching to X4. A higher stability of viral tropism (88%) during ARV was previously reported [15] in a smaller cohort, with 12% switching in either direction. Interestingly, a successful amplification result could be obtained for only 33 out of 53 subjects, with respect to 100% of our cohort. Soulié et al.correlated the DNA and plasma RNA genotypes of fully responding patients after 2 years of therapy and found a stable genotype in 92.9% of subjects [16]. In their study, the switch in viral tropism is demonstrated by looking at the difference in the viral genotypes in the samples of cellular DNA during successful therapy and plasma RNA, collected at the time of detectable viraemia. In our study, the switch in viral tropism was assessed using the cellular DNA samples collected longitudinally at different times from patients who fully responded to ARV, and therefore, the results can be difficult to compare. Furthermore, it has been previously demonstrated that the viral genotypes of the strains stored in DNA compared to that detectable in the plasma during replication may be different [22]; this finding casting doubts about the reliability of the results of Soulie’s study.

The stability of tropism remained significant in the second period of observation (T0 to T2 p = 0.0003); in the second follow-up interval, 16% of the subjects switched, and a correlation between a switch to X4 and a decrease in the CD4 count was observed. No differences in relation to the therapy administered at the time of follow-up were observed in the different groups.

The emergence of X4 viruses generally occurs in the later stages of disease [7-9], and it is associated with low CD4 T-cell counts. Several studies have demonstrated a higher prevalence of X4 variants in patients exposed to antiretroviral drugs than in drug-naïve individuals [10,11]. Delobel et al. suggested that the emergence of X4 variants on HAART may be due to the preferential implication of X4 variants in cellular reservoirs, either through a progressive replenishment of the pool of resting memory CD4+ T cells by cells derived from long-lived naive CD4+ T cells harboring archival X4 species, or through the preferential residual replication of X4 viruses on HAART. About the relation among this potentially selective replication of strains with different tropism in various immuno-virological conditions, we investigated the residual viral replication in our patients.

Among 219 successfully treated subjects, 38% reached complete control of viral replication, defined as an HIV RNA copy number under the detection limit of 2.5 copies/ml, by the first follow-up. This rate is comparable with those of other studies reporting success rates of 36-40%, but it is lower than that of another cohort study reporting a success rate 60% [20,23]. A significant persistence of the acquired lowest RNA level was observed (p= 0.002). The patients harbouring an X4 strain at T1 were less able to reach this virologic success at T2 (p= 0.0076). Of note, 40% of patients stable R5 or switching from R5 with respect to 10% of stable X4 or switching from X4 revealed HIV RNA <2,5 copies/ml at T2. Confirming this result, in the subgroup of 51 subjects with evaluations at all three time points, patients with stable R5-tropic strain or with R5 in two out of three determinations demonstrated a greater control in plasma replication than stable or prevalent X4-tropic patients; a 24% success rate in terms of complete suppression of residual replication in stable R5-tropic was observed, while the seven subjects with stable X4 never showed controlled replication at a level below 2.5 copies/ml. Sixty-two percent the subjects with plasma RNA levels below 2,5 copies/ml were stable R5-tropic patients.

These results allow us to suggest that the total amount of time spent with the R5 condition appears to be relevant for reaching the best control of plasma viraemia, rather than the current tropism condition. To our knowledge, no data are available on this matter, making this observation the first demonstration of a correlation between an R5 genotype and a better virologic control during follow-up in treatment responder patients.

In our cohort, 73 subjects (33.4%) had an X4 strain in their PBMCs at the first follow-up after treatment initiation; the absolute CD4 count and the CD4 percentage were higher in R5- than in X4-tropic patients at baseline and at the follow-up time points. The T0 absolute CD4 count was particularly high in R5-tropic patients, reaching statistical significance with respect to X4 subjects (p= 0.0216). On the contrary, no significant difference was found in the follow-up assessments at different time points in the recovery of CD4+ cells in patients harbouring an R5- or X4-tropic virus. This result confirms those obtained in previous studies showing no differences in the virological treatment response between patients harbouring an X4- or R5-tropic virus, although a poor recovery of CD4 cells has been shown in patients with an X4-tropic virus at baseline or after an R5 to X4 virus switch [7,11-13,24]. Nevertheless, in our cohort the mean CD4 count increased in the interval T1 to T2, and this increase was significantly higher patients harbouring R5 coreceptor tropism than for X4 tropism (p = 0.0497). This finding suggests a possible difference in the long-term course of this disease. Some biases of the study should be considered. First of all, since viral DNA sequenced from PBMCs may be a reflection of HIV archived at different stages of infection, changes in viral tropism detected in our study may be caused by a sampling issue or by technical reasons in consequence of the use of population sequencing rather than viral evolution. To avoid or minimize these biases we repeated twice all sequences with a false positive rate surrounding the cut-off values. Additionally, it was not possible to obtain an analysis of the tropism for all patients at all time points studied. However, this study analysed numerous viro-immunological parameters, and a large number of patients was evaluated at individual time points. In 51 patients, it was possible to evaluate the various parameters throughout the course of treatment.

## Conclusions

In successfully treated naïve subjects, the tropism of archived virus was revealed to be stable, but with a substantial rate (15-18%) of subjects switching from one type to the other over time, despite a viraemia <50 copies/ml. An R5 tropism and its stability on follow-up were related to achieving and maintaining very low levels (less than 2.5 copies/ml of plasma) of viral replication in treatment responder patients, suggesting a relation among viral tropism and response to treatment in the long term.

## Competing interests

The authors declare that they have no competing interests.

## Authors’ contributions

SGP designed and coordinated the study, supervised the laboratory experiments, collected the data, interpreted the findings, and wrote the paper; SA performed the laboratory experiments; CM interpreted the data and performed the statistical analysis; RS helped to design the study, managed the patients, and collected the samples; MC managed the patients, collected the samples and helped to interpret the findings and write the paper; RF managed the patients and collected the samples; VM managed the patients and collected the samples; SP managed the patients and collected the samples; MB helped to interpret the findings and write the paper; CB performed the laboratory experiments; SB performed the laboratory experiments; LS helped to interpret the findings and write the paper; MA helped to interpret the findings and write the paper; and GP designed the study and helped to interpret the findings and write the paper. All authors read and approved the final manuscript. SGP, RS, MC, RF, VM, SP, and GP are members of CAVeAT (Cohort of Amici Venetian for Antiretroviral Treatment).

## Pre-publication history

The pre-publication history for this paper can be accessed here:

http://www.biomedcentral.com/1471-2334/13/314/prepub

## References

[B1] Choe H Farzan M Sun Y Sullivan N Rollins B Ponath PD Wu L Mackay CR LaRosa G Newman W Gerard N Gerard C Sodroski J The beta-chemokine receptors CCR3 and CCR5 facilitate infection by primary HIV-1 isolates Cell 1996 85 1135 1148 10.1016/S0092-8674(00)81313-6 8674119

[B2] Dragic T Litwin W Allaway GP Martin SR Huang Y Nagashima KA Cayanan C Maddon PJ Koup RA Moore JP Paxton WA HIV-1 entry into CD4+ cells ismediated by the chemokine receptor CC-CKR-5 Nature 1996 381 667 673 10.1038/381667a0 8649512

[B3] Deng H Liu R Ellmeier W Choe S Unutmaz D Burkhart M Di Marzio P Marmon S Sutton RE Hill CM Davis CB Peiper SC Schall TJ Littman DR Landau NR Identification of a major co-receptor for primary isolates of HIV-1 Nature 1996 381 661 666 10.1038/381661a0 8649511

[B4] Alkhatib G Combadiere C Broder CC Feng Y Kennedy PE Murphy PM Berger EA CC CKR5: a RANTES, MIP-1alpha, MIP-1beta receptor as a fusion cofactor for macrophage-tropic HIV-1 Science 1996 272 1955 1958 10.1126/science.272.5270.1955 8658171

[B5] Feng Y Broder CC Kennedy PE Berger EA HIV-1 entry cofactor: functional cDNA cloning of a seven-transmembrane, G protein-coupled receptor Science 1996 272 872 877 10.1126/science.272.5263.872 8629022

[B6] Poveda E Briz V Quiñones-Mateu M Soriano V HIV tropism: diagnostic tools and implications for disease progression and treatment with entry inhibitors AIDS 2006 20 1359 1367 10.1097/01.aids.0000233569.74769.69 16791010

[B7] Waters L Mandalia S Randell P Wildfire A Gazzard B Moyle G The impact of HIV tropism on decreases in CD4 cell count, clinical progression, and subsequent response to a first antiretroviral therapy regimen Clin Infect Dis 2008 46 1617 1623 10.1086/587660 18419499

[B8] Schuitemaker H Koot M Kootstra NA Dercksen MW de Goede RE van Steenwijk RP Lange JM Schattenkerk JK Miedema F Tersmette M Biological phenotype of HIV type 1 clones at different stages of infection: progression of disease is associated with a shift from mono-cytotropic to T-cell-tropic virus population J Virol 1992 66 1354 1360 1738194 10.1128/jvi.66.3.1354-1360.1992 PMC240857

[B9] Hunt PW Harrigan R Huang W Bates M Williamson DW McCune JM Price RW Spudich SS Lampiris H Hoh R Leigler T Martin JN Deeks SG Prevalence of CXCR4 tropism among antiretroviral-treated HIV-1infected patients with detectable viremia J Infect Dis 2006 194 926 930 10.1086/507312 16960780

[B10] Johnston ER Zijenah LS Mutetwa S Kantor R Kittinunvorakoon C Katzenstein DA High frequency of syncytium-inducing and CXCR4-tropic viruses among HIV type 1 subtype C-infected patients receiving antiretroviral treatment J Virol 2003 77 7682 7688 10.1128/JVI.77.13.7682-7688.2003 12805470 PMC164829

[B11] Delobel P Sandres-Sauné K Cazabat M Pasquier C Marchou B Massip P Izopet J R5 to X4 switch of the predominant HIV-1 population in cellular reservoirs during effective highly active antiretroviral therapy J Acquir Immune Defic Syndr 2005 38 382 392 10.1097/01.qai.0000152835.17747.47 15764954

[B12] Bratt G Karlsson A Leandersson AC Albert J Wahren B Sandström E Treatment history and baseline viral load, but not viral tropism or CCR-5 genotype, influence prolonged antiviral efficacy of highly active antiretroviral treatment AIDS 1998 12 2193 2202 10.1097/00002030-199816000-00015 9833861

[B13] Seclén E Soriano V González MM Martín-Carbonero L Gellermann H Distel M Kadus W Poveda E Impact of baseline HIV-1 tropism on viral response and CD4 cell count gains in HIV-infected patients receiving first-line antiretroviral therapy J Infect Dis 2011 204 139 144 10.1093/infdis/jir218 21628668 PMC3307159

[B14] Soulié C Fourati S Lambert-Niclot S Malet I Wirden M Tubiana R Valantin MA Katlama C Calvez V Marcelin AG Factors associated with proviral DNA HIV-1 tropism in antiretroviral therapy-treated patients with fully suppressed plasma HIV viral load: implications for the clinical use of CCR5 antagonists J Antimicrob Chemother 2010 65 749 751 10.1093/jac/dkq029 20150182

[B15] Seclén E Del Mar González M De Mendoza C Soriano V Poveda E Dynamics of HIV tropism under suppressive antiretroviral therapy: implications for tropism testing in subjects with undetectable viraemia J Antimicrob Chemother 2010 65 1493 1496 10.1093/jac/dkq156 20488982

[B16] Soulie C Lambert-Niclot S Wirden M Simon A Valantin MA Fourati S Tubiana R Katlama C Calvez V Marcelin AG Low frequency of HIV-1 tropism evolution in patients successfully treated for at least 2 years AIDS 2011 25 537 539 10.1097/QAD.0b013e32834345d3 21139487

[B17] Saracino A Monno L Cibelli DC Punzi G Brindicci G Ladisa N Tartaglia A Lagioia A Angarano G Co-receptor switch during HAART is independent of virological success J Med Virol 2009 81 2036 2044 10.1002/jmv.21598 19856465

[B18] Waters LJ Scourfield AT Marcano M Gazzard BG Bower M Nelson M Stebbing J The evolution of coreceptor tropism in HIV-infected patients interrupting suppressive antiretroviral therapy Clin Infect Dis 2011 52 671 673 10.1093/cid/ciq198 21292672

[B19] Sarmati L Parisi SG Andreoni C Nicastri E Buonomini AR Boldrin C Dori L Montano M Tommasi C Andreis S Vullo V Palù G Andreoni M Switching of inferred tropism caused by HIV during interruption of antiretroviral therapy J Clin Microbiol 2010 48 2586 2588 10.1128/JCM.02125-09 20484604 PMC2897474

[B20] Parisi SG Andreis S Mengoli C Scaggiante R Ferretto R Manfrin V Cruciani M Giobbia M Boldrin C Basso M Andreoni M Palù G Sarmati L Baseline cellular HIV DNA load predicts HIV DNA decline and residual HIV plasma levels during effective antiretroviral therapy J Clin Microbiol 2012 50 258 263 10.1128/JCM.06022-11 22135262 PMC3264143

[B21] Nicastri E Palmisano L Sarmati L D’Ettorre G Parisi S Andreotti M Buonomini A Pirillo FM Narciso P Bellagamba R Vullo V Montano M Di Perri G Andreoni M HIV-1 residual viremia and proviral DNA in patients with suppressed plasma viralLoad (<400 HIV-RNA cp/ml) during different antiretroviral regimens Curr HIV Res 2008 6 261 266 10.2174/157016208784325010 18473790

[B22] Parisi SG Andreoni C Sarmati L Boldrin C Buonomini AR Andreis S Scaggiante R Cruciani M Bosco O Manfrin V D’Ettorre G Mengoli C Vullo V Palù G Andreoni M HIV coreceptor tropism in paired plasma, peripheral blood mononuclear cell and cerebrospinal fluid isolates from antiretroviral naïve subjects J Clin Microbiol 2011 49 1441 1445 10.1128/JCM.02564-10 21367995 PMC3122871

[B23] Gianotti N Galli L Racca S Salpietro S Cossarini F Spagnuolo V Barda B Canducci F Clementi M Lazzarin A Castagna A Residual viraemia does not influence 1 year virological rebound in HIV-infected patients with HIV RNA persistently below 50 copies/mL J Antimicrob Chemother 2012 67 213 217 10.1093/jac/dkr422 21987242

[B24] Charpentier C Joly V Larrouy L Fagard C Visseaux B de Verdiere NC Raffi F Yeni P Descamps D on behalf of the ANRS 130 APOLLO Trial Study Group Role and evolution of viral tropism in patients with advanced HIV disease receiving intensified initial regimen in the ANRS 130 APOLLO trial J Antimicrob Chemother 2013 68 690 696 10.1093/jac/dks455 23152480

